# How to handle occupational well-being of critical care workers. A lesson from the pandemic: Nicola Magnavita

**DOI:** 10.1093/eurpub/ckac131.087

**Published:** 2022-10-25

**Authors:** N Magnavita, PM Soave, M Antonelli

**Affiliations:** 1 Postgraduate School of Occupational Health, Università Cattolica del Sacro Cuore, Rome, Italy; 2 Department of Woman, Child and Public Health Sciences, Fondazione Policlinico Gemelli IRCCS, Rome, Italy; 3 Department of Emergency, Anesthesiology and Resuscitation Sciences, Fondazione Policlinico Gemelli IRCCS, Rome, Italy

## Abstract

**Background:**

The PSIC study (Prospective Study of Intensivists and COVID-19) monitored the intensivists working in one of the two COVID-19 hub hospitals in Central Italy over 2 years from April 2020. This study showed how mental health varies in relation to the stressors posed by the different pandemic phases.

**Methods:**

In 4 surveys corresponding to the 4 pandemic waves, the intensivists were invited to indicate changes in work activity and measure their state of mental health using standardized questionnaires administered via SurveyMonkey.

**Results:**

During the pandemic there was a change in occupational stressors that led to insomnia, anxiety, depression, burnout, job dissatisfaction, unhappiness and intention to quit. The predominant stressors in the first wave were fear of unprotected exposure, distrust of safety measures, and compassion fatigue from having to inform relatives of the adverse outcome of treatment. In the second and third waves the workload, the monotony due to always following only one type of patient, the isolation, and the lack of time to meditate were the more relevant factors. The fourth wave added the stress deriving from interacting with anti-vax patients

**Conclusions:**

Specific prevention strategies have been developed and applied for each of the stress factors identified. Excessive workload and lack of time for meditation originated from lack of staff were remedied with extraordinary temporary hires. The management of compassion fatigue and relations with anti-vax people were addressed with specific policies and training. The monotony and isolation in COVID-19 wards can only be resolved through employee turnover in ordinary departments. Organizational and financial efforts are necessary to protect the health of intensivists during a pandemic.

**Key messages:**

• Monitoring of critical care workers during the pandemic waves indicated the preventive measures necessary to ensure their mental health and quality of care.

• Protecting healthcare workers is a priority.

